# Causality Dilemma: Reassessing the Role of Interferon Signature Genes in Human Disease

**DOI:** 10.3390/biology15212018

**Published:** 2026-11-06

**Authors:** Ryan G. Gaudet, Hamna Shahnawaz, Dominic A. Ritacco

**Affiliations:** Department of Microbiology & Immunology, Columbia University, Irving Medical Center, New York, NY 10032, USAdar2213@cumc.columbia.edu (D.A.R.)

**Keywords:** interferon, interferon-stimulated genes, mitochondria, inflammation, immunopathology, host–pathogen interaction, cell-intrinsic immunity

## Abstract

Interferons (IFNs) are synonymous with mobilizing cell-intrinsic defenses against microbial infection through the induction of interferon-stimulated genes (ISGs). Omics-based profiling has revealed that ISGs also dominate the transcriptional signature of a wide range of human disease conditions, including cancer, autoimmune and autoinflammatory disorders, and even normal aging. The ubiquity of the IFN signature means it is often interpreted as a biomarker for upstream inflammation, with little consideration given to how persistent ISG induction might influence downstream cellular and tissue physiology. However, the identification of endogenous targets for a growing number of ISG-encoded effectors has broadened our appreciation for the function of these genes—both in terms of how self-defense is achieved but also for the potential implications emanating from their prolonged induction. The same host-directed mechanisms that provide broad antimicrobial protection can, depending on context, either preserve tissue homeostasis or contribute to chronic inflammation and tissue dysfunction. In this perspective, we revisit the longstanding question of IFN causality in disease and argue that ISGs should be viewed as active participants in pathogenesis rather than passive markers of inflammatory activity. By examining disease-specific ISG expression patterns and synthesizing emerging evidence for the endogenous functions of ISGs, many of which converge on mitochondrial regulation, we propose a framework in which the composition of an IFN signature associated with a diseased tissue plays a major role in dictating whether inflammation is resolved or perpetuated.

## Simple Summary

This article presents a timely argument that interferon-stimulated genes (ISGs), which are often interpreted as biomarkers of inflammation, should be viewed as active contributors to disease pathogenesis. By highlighting novel tissue-specific expression patterns across diseases and emerging evidence for endogenous, homeostatic functions for ISGs, this perspective provides a framework in which the composition of an IFN signature shapes the outcome of an inflammatory response.

## 1. Introduction

The innate immune system deploys a remarkable arsenal of effectors for cell-intrinsic defense against infection. A defining feature of this response is its inducibility: Upon sensing danger, cells rapidly activate hundreds of antimicrobial effectors, many of which are transcriptionally elicited by activating cytokines, the most powerful of which are the interferons (IFNs). By sequence homology, IFNs are classified into three families: Type I IFNs (IFNαs, IFN-β, IFN-ε, IFN-κ, and IFN-ω); Type II IFN (IFN-γ); and Type III IFNs (IFN-λs). The distinct biological roles of each IFN type reflect differences in their modes of induction, cellular sources, and target cell populations (for recent reviews, see [1,2]). Despite these differences, all IFN classes elicit effector responses through the induction of interferon-stimulated genes (ISGs), collectively referred to as the ‘interferome’ [3].

Because IFNs are indispensable for host defense, studies of ISG biology have historically focused on how the proteins encoded by these genes directly combat pathogens [4,5,6]. This work has established a diverse collection of antiviral, antibacterial, and antiparasitic effectors that act at virtually every stage of the infectious process. However, IFNs are not produced exclusively in response to infection. Rather, they serve as broader signals of tissue stress and disrupted homeostasis and are induced by a wide range of endogenous and exogenous insults. Consequently, ISG signatures are a pervasive feature of many inflammatory disorders, including autoimmune disease, neurodegeneration, metabolic dysfunction, cancer, and aging.

Despite their ubiquity across human disease, the biological significance of persistent ISG induction remains poorly understood. In most settings, IFN signatures are interpreted as biomarkers of inflammation rather than active contributors to pathology. This view assumes that ISG induction largely reflects upstream inflammatory processes and does not substantially influence disease progression itself. Increasing evidence, however, suggests that this interpretation may be incomplete. A growing number of ISGs now appear to protect the host not only by directly targeting pathogens, but by rewiring endogenous cellular pathways. These host-directed activities include regulating metabolism, implementing organelle quality control, modifying membrane dynamics, influencing cell death pathways, and shaping inflammatory signaling networks. While such mechanisms can provide broad antimicrobial protection, they pose a substantial risk for self-harm and create opportunities for unintended effects on tissue function when IFN signaling becomes chronic or dysregulated. As a result, the biological effects of many ISGs are likely to manifest in inflamed tissues even in the absence of microbial infection.

In this perspective, we argue that ISGs should be viewed not only as collections of antimicrobial or inflammatory signaling effectors, but as regulators of tissue physiology whose endogenous functions can directly influence disease outcome. By examining emerging examples in which individual ISGs either promote pathology or preserve homeostasis, we propose a conceptual framework in which the specific composition of an IFN signature actively shapes disease pathogenesis rather than merely serving as a downstream marker of inflammation.

## 2. Emerging Patterns for ISG Signatures Across Tissues and Disease Contexts

On the basis of early studies enlisting bulk sequencing or microarray profiling of IFN-primed cell lines, Type I, II, and III IFNs were initially assumed to induce largely overlapping transcriptional programs across cell types, a phenomenon reflected by the global induction of canonical ISGs in a variety of immune disorders and disease states [7,8]. However, more recent single-cell analysis has revealed extensive cell type-, tissue-, and organ-specific heterogeneity in how cells respond to interferons, with stimuli-specific induction of ISGs regulated through epigenetic and post-transcriptional mechanisms, which collectively shape the disease footprints associated with specific ISG signatures [9,10,11,12]. To gain further insight into how the composition of ISG signatures differs across disease and tissue states, we assembled an updated compendium of 2201 human interferon-regulated genes based on metanalysis of 14 studies profiling human cells stimulated with recombinant type I or type II IFN [10,13,14,15,16,17,18,19,20,21,22,23,24,25] (refer to Supplementary File S1 for study details). To account for the influence of differing analytical pipelines across datasets, we computed differential expression with a single threshold for every study where per-sample data were available, and required each gene to be induced in at least two independent studies of the same IFN class.

**Figure 1:**
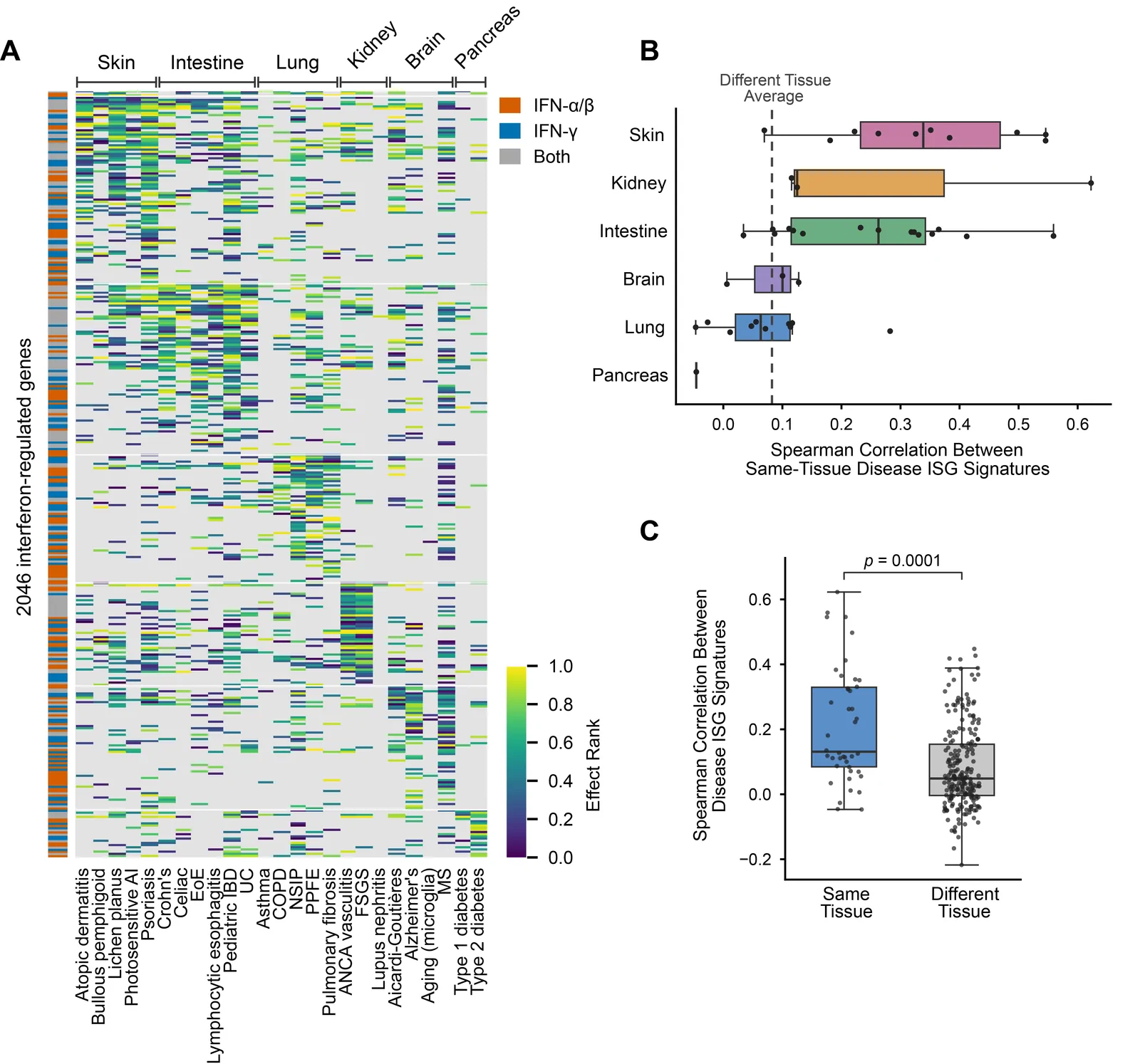
Context-specific ISG signatures observed across tissue and disease states. To define the set of ISGs eligible for disease analysis, we compiled 14 publicly available transcriptomic datasets (Supplementary Table S1 for study details) in which healthy human cells or tissues were stimulated with one or more interferon subtypes (type I or type II). Datasets spanned structural cell types and immune cell types across skin, lung, intestinal, vascular, and blood compartments. Differential expression was computed or obtained per dataset by the method best suited to the available data (per-dataset methods depicted in Supplementary Table S1). For multi-timepoint datasets the most mature IFN response (typically 6–12 h) was used with each dataset counted once per interferon class. A gene was called induced if log_2_FC ≥ 0.5 in at least two independent datasets of the same interferon class and adjusted *p* < 0.05 (Benjamini–Hochberg) yielding our final interferome composed of 2201 human genes (full gene list in Supplementary Table S2). This interferome was then intersected with disease-vs-healthy differential-expression results for 25 inflammatory or autoimmune diseases. Fourteen of these disease signatures were computed from publicly available per-sample data, with DESeq2 for count data, or limma for microarray and normalized expression data; the remaining 11 use the authors’ published tables. (See Supplementary Table S3 for study descriptions). An individual ISG was counted as overexpressed in a disease if the effect rank > 0.2 and, where a significance value was reported, adjusted *p*/FDR < 0.05 (a full list of the effect ranks for each ISG is available in Supplementary Table S4). To minimize variability across studies, disease correlations are reported as effect rank, the percentile rank of its log_2_FC among all genes upregulated in that study, which is robust to heterogeneity in the methods used across studies. Genes not reported as induced in a given disease are shown as grey. (**A**) Total number of ISGs (*n* = 2046) identified as upregulated in at least one disease condition. Disease is listed on the bottom; the afflicted tissue is listed on the top, sampled tissue is given in Supplementary Table S2. (**B**) Diseases of the same tissue share more similar ISG signatures than diseases of different tissues. Spearman correlation of interferome gene effect ranks for ISGs measured across two studies in either the same or different tissue site. Each point represents one pair of diseases with pairs grouped by whether the two diseases affect the same tissue or different tissue. Boxes show the median and interquartile range; whiskers extend to 1.5 times the interquartile range. (**C**) Cumulative analysis of Spearman Correlation of the ISG signatures from diseases within same-tissue pairs compared to different-tissue pairs, *p* = 0.001, one-sided permutation test with 10,000 shuffles of tissue labels. IBD, inflammatory bowel disease; UC, ulcerative colitis; EoE, eosinophilic esophagitis; COPD, chronic obstructive pulmonary disease; PPFE, pleuroparenchymal fibroelastosis; NSIP, nonspecific interstitial pneumonia; ANCA, antineutrophil cytoplasmic antibodies. Plots in (**A**,**B**) are presented on the same scale. See Supplementary Table S5 for software details.

Our updated catalogue of ISGs was then queried with differentially expressed gene sets across 25 inflammatory or autoimmune-related diseases to identify overrepresented ISGs for each tissue and condition, identifying 2046 genes meeting statistical cutoff in at least one disease dataset [26,27,28,29,30,31,32,33,34,35,36,37,38,39,40,41,42,43,44,45,46,47,48] (Figure 1A, Supplementary File S1). Whereas some canonical ISGs were significantly upregulated across several contexts, many ISGs clustered to specific types of diseased tissue. Consistent with this, the affected tissue explained 33% of the variance between disease ISG signatures (PERMANOVA, *p* = 0.0001), and diseases of the same tissue shared more similar ISG signatures than when compared to diseases affecting a different tissue site, especially for in skin, intestine, lung and kidney (Figure 1B,C). Notably, ISGs with functional relationships, for example *GBP1* and *APOL3* encode effectors which converge on bacterial membranes [49] or *PSMB8* and *TAP1* which encode antigen processing machinery, exhibit similar disease association profiles, suggesting that ISGs clustering to a specific disease or tissue may exhibit genetic interactions that alter pathogenesis. These findings imply that IFN signatures are not generic indicators of inflammation, but rather highly contextualized programs whose composition may encode important information about tissue state and disease trajectory. A limitation to this type of analysis is that because of ethical and technical difficulties in tissue acquisition, our human interferon-stimulated gene catalogue is collated from studies enlisting IFN-activated primary cells, organoids, blood immune cells or isolated cell lines rather than measured within native human tissues that preserve microenvironmental cues that could dictate gene inducibility. Thus, it is unclear whether overexpressed ISGs represent baseline programs normally induced in these tissues, or if they are shaped by disease-associated signals and represent potentially pathogenic alterations to the baseline signature of a given site. In addition, disease signatures derived from bulk tissue transcriptomics likely reflect changes in cellular composition as well as induction within resident cells, as the two cannot be separated within these data. Both of these considerations represent important avenues for future research.

## 3. ISGs as Active Drivers of Disease Outcome

A central unresolved question in how IFNs associate with modern human diseases is whether an observed ISG signature merely reflects ongoing inflammation or actively contributes to disease progression. The latter possibility has received comparatively little attention. This is partly because many ISGs were initially discovered and characterized for their antimicrobial functions, creating a conceptual framework in which their primary importance was assumed to lie in pathogen restriction. However, a growing body of evidence suggests that in the inflammatory niche where IFNs are concentrated, numerous ISGs influence host physiology independently of infection. In these contexts, their activities can either amplify inflammatory pathology or preserve tissue homeostasis, depending on the cellular environment, upstream stimuli, and endogenous pathways targeted.

The examples discussed below have leveraged mouse models, cellular experiments, and human genetic association studies to provide evidence linking the induction of specific ISG-encoded effectors directly to disease progression. We focus specifically on diseases involving immunopathology rather than malignancies which is beyond the scope of this perspective; for discussions of the dual nature of IFNs in cancer progression the reader is referred to several recent reviews [50,51]. These examples illustrate several recurring themes through which ISGs shape disease biology, including metabolic rewiring, regulation of cell death pathways, control of organelle homeostasis, and modulation of inflammatory signaling networks. Collectively, these studies challenge the prevailing view of IFN signatures as passive disease markers and instead support a model in which ISGs function as active determinants of tissue health and disease.

### 3.1. ISGs as Mediators of Disease Pathology

Tissues exhibit differential susceptibility to IFN-driven tissue damage, with the brain being particularly sensitive to the effects of Type II IFN. Animal models have implicated IFN-γ in multiple neurodegenerative conditions, including neurodegenerative models of MS [52], Alzheimer’s disease [53], Parkinson’s disease [54], and white matter aging [55]. However, the mechanisms directly linking IFN-γ signaling to neuronal dysfunction remain poorly understood. Recent work has identified neuron-intrinsic ISGs as direct mediators of these effects. Woo et al., (2025) demonstrated that IFN-γ-dependent induction of *PSMB8*, which encodes the catalytic component of the immunoproteasome, disturbed neuronal metabolism by altering proteasomal balance, leading to enhanced glycolysis, oxidative injury and ferroptosis in a mouse model of experimental autoimmune encephalomyelitis (EAE) [56]. Under normal inflammatory conditions, transient replacement of constitutive proteasomal subunits with immunoproteasome components facilitate the clearance of damaged proteins, modulate inflammatory signaling, and enhance MHC-I antigen presentation [57]. The de novo formation of the immunoproteasome complex induced by IFN-γ is normally transient in nature and exhibits a much shorter half-life compared to constitutive versions, highlighting the imperative to tightly control its activity [58]. Chronic IFN-γ signaling, however, may disrupt this balance as persistent induction of the immunoproteasome in CNS-resident cells like astrocytes, microglia, and specific neuronal populations is observed across a plethora of neurological disorders, including ischemic stroke, Alzheimer’s disease, Parkinson’s disease, Huntington’s disease, and epilepsy [57,59]. Thus, chronic induction of proteasome-associated ISGs may represent a common mechanism linking neuroinflammation to metabolic dysfunction, proteotoxic stress and neuronal loss.

A second example of pathological host remodeling in the brain is provided by IFITM3. Traditionally viewed as an antiviral restriction factor that prevents viral escape from endosomes [60,61,62], IFITM3 has recently been implicated in Alzheimer’s disease pathogenesis through a completely different mechanism. During neuroinflammation, IFITM3 associates with the host γ-secretase complex, enhancing cleavage of amyloid precursor protein and increasing production of amyloid-β peptides [63]. Because IFITM3 expression is induced by both Type I and Type II IFNs and increases with age, this interaction establishes a direct molecular link between inflammatory signaling and amyloid plaque formation. Moreover, in immortalized murine microglia, sensing of amyloid deposits activates a cGAS-STING-IFITM3 signaling axis that further amplifies *IFITM3* expression, creating a potentially pathogenic feed-forward loop [64]. Human genetic studies reinforce this connection, as the variant rs10751647 associated with elevated *IFITM3* levels correlates with cognitive decline [65]. This effect is recapitulated in animal models where experimental overexpression exacerbates anxiety and neurodegeneration in septic mice [66]. Together, *PSMB8* and *IFITM3* illustrate how ISGs that evolved to protect the organism from infection can become pathogenic when their effects on endogenous metabolic and proteolytic pathways are sustained, particularly in vulnerable tissue like the brain.

Another mechanism through which ISGs contribute to disease involves licensing inflammatory cell death pathways. This phenomenon is particularly evident in barrier tissues where immune-mediated pathogen control must be carefully balanced against the need to preserve tissue integrity. The intestine provides a striking example of this trade-off. Although IFN signaling has long been implicated in the pathogenesis of inflammatory bowel disease (IBD), its precise role has proven difficult to resolve across experimental models and patient populations. In this context, much of the focus has been on exploiting the IFN signature as a clinical biomarker that predicts disease course and therapeutic response [67,68]. However, these associations provide limited insight into how IFN responses influence disease progression. A more mechanistic focus on the tissue-intrinsic functions of individual ISGs may help resolve this ambiguity by revealing how specific IFN-induced effectors shape intestinal inflammation and tissue injury. For example, Jena et al. (2024) identified a mechanism through which Type III IFNs directly inhibit intestinal repair [69]. In a mouse model of intestinal injury, IFN-λ2/λ3 signaling induced expression of the nucleic acid sensor ZBP1 in intestinal epithelial cells, licensing recognition of endogenous Z-DNA structures generated during tissue damage. ZBP1 activation subsequently triggered caspase-8- and GSDMC-dependent pyroptosis, thereby limiting epithelial regeneration. Consistent with these findings, increased expression of Type III IFNs, ZBP1, cleaved caspase-8, and GSDMC was observed in human biopsies from patients with IBD, supporting a potential role for this pathway in human disease [69].

*ZBP1* also illustrates the highly context-dependent nature of ISG function. Although induction of *ZBP1* is a common feature across diverse inflammatory settings, studies in mice have revealed how the downstream consequences of ZBP1 engagement vary considerably depending on the tissue involved, the inducing stimulus and the nucleic acid species detected. In intestinal stem cells lacking SETDB1-mediated repression of endogenous retroviruses, Type I IFN promotes ZBP1-dependent necroptosis rather than pyroptosis, resulting in loss of epithelial integrity and impaired intestinal homeostasis [70,71]. Similar ZBP1-dependent necroptotic programs operate in virus-infected lung epithelial cells, whereas in steatotic liver ischemia–reperfusion injury, elevated reactive oxygen species levels trigger ZBP1-dependent apoptosis independently of nucleic acid sensing, contributing to liver damage and allograft dysfunction [72,73,74]. These findings demonstrate that the biological consequences emanating from ISG induction cannot be inferred solely from its presence within a transcriptional signature. Instead, its effects emerge from the interactions between tissue environment, upstream stress signals, and downstream pathways available for activation.

Beyond directly damaging tissues or inducing cell death, ISGs can amplify other inflammatory signaling pathways that contribute to disease progression. Systemic lupus erythematosus (SLE), a disorder characterized by a prominent type I IFN signature [75,76,77,78], provides a compelling example of this phenomenon. In SLE, defective clearance of mitochondria-containing erythrocytes promotes cGAS-STING activation in monocytes, which is thought to contribute to the sustained ISG expression observed in patients [79]. These monocytes exhibit a distinctive hyperinflammatory phenotype characterized by simultaneous expression of ISGs and secretion of mature IL-1β despite lacking canonical inflammasome activation [80]. Recent work identified an ISG encoding the antiviral effector myxovirus-resistant protein 1 (MxA) as a key mediator of this process. Although MxA was originally characterized for its ability to block nuclear trafficking of viral proteins, it also redirects host IL-1β into the trans-Golgi network, enabling unconventional cytokine secretion [81]. Thus, a protein that likely evolved to interfere with viral transport is repurposed under inflammatory conditions to amplify endogenous cytokine signaling. More broadly, these findings suggest that by rewiring inflammatory pathways, ISGs may explain how IL-1β secretion is sustained in other IFN-associated conditions, including experimental autoimmune encephalitis and aging [82,83].

Inflammasome activation represents another point at which ISGs intersect with inflammatory disease. Type I and II IFNs influence the NLRP3 inflammasome through multiple mechanisms, including the induction of core inflammasome components like the gasdermins (GSDMs) or regulators like the p65 guanylate binding proteins (GBPs) [84,85]. However, other important regulatory effects may arise from ISGs whose primary functions lie outside of canonical inflammasome biology. One such example is *CMPK2.* Although CMPK2 was initially characterized as an antiviral nucleotide kinase involved in the synthesis of 3′-deoxy-3′,4′-didehydro-cytidine triphosphate (ddhCTP), a chain terminator for RNA-dependent RNA Polymerase (RDRP) [86,87,88], it also performs the rate-limiting step in generating deoxynucleotide precursors required for mitochondrial DNA synthesis [89]. Newly synthesized mtDNA is highly susceptible to oxidation and is preferentially released into the cytosol via membrane pores that open after exposure to NLRP3 activators. In the absence of *CMPK2*, mtDNA is not released, and NLRP3 is refractory to its agonists in mouse models [89]. Consequently, CMPK2 acts as a critical licensing factor for inflammasome activation. Consistent with this role, elevated *CMPK2* expression in immune cells is linked to pathogenic type I IFN responses observed in SLE patients and potentially across other disease contexts where additional signals trigger its spurious expression [90,91]. For example, chronically elevated glucocorticoids induce sustained *Cmpk2* expression in the bone, and inhibiting *Cmpk2* in stem cells and proteoblasts alleviated glucocorticoid-induced senescence by restoring mitochondrial function [92]. Together, these studies suggest that chronic induction of *CMPK2* and other NLRP3-interacting ISGs may lower the threshold for inflammasome-driven pathology.

### 3.2. ISGs as Guardians of Tissue Homeostasis

The pathogenic activities of ISGs discussed above represent only one side of the relationship between IFN signaling and disease. Given the indispensable role of IFNs in host defense, it would be surprising if persistent ISG expression universally promoted tissue injury. Indeed, many ISGs encode proteins that preserve cellular homeostasis, limit excessive inflammation, or facilitate recovery from stress. In these contexts, antimicrobial activities directed against pathogens often reflect broader abilities to monitor and regulate endogenous cellular processes. Consequently, the net effect of an IFN signature depends not only on the presence of pathogenic effectors but also on the balance of protective ISGs that maintain tissue integrity.

A recurring theme amongst protective ISGs is the regulation of organelle quality control pathways. Many antimicrobial effectors target host organelles that are remodeled during infection, but several of these same ISGs have also been observed targeting endogenous organelles during non-infectious stress where they function to preserve cellular fitness. The 47-kilodalton immunity-related GTPases (IRGs) provide a striking example of this principle. In mice, the IRG family comprises more than twenty IFN-inducible genes that protect against a remarkably diverse collection of intracellular pathogens by regulating membrane trafficking and promoting fusion of pathogen-containing compartments with lysosomes [93,94]. Humans, in contrast, retain a single family member, IRGM, which is required for IFN-γ-mediated control of *Mycobacterium tuberculosis* [95,96]. Unexpectedly, the protective effects of IRGM are mediated largely through regulation of endogenous organelles rather than direct pathogen targeting. IRGM controls mitochondrial fission and promotes autophagy, two housekeeping processes that are essential for maintaining cellular homeostasis under stress [97]. Consistent with this role, genetic variants in IRGM have long been associated with increased susceptibility to Crohn’s disease [98,99,100]. Similar functions are observed in mice, where Irgm1 regulates both autophagosome turnover and mitochondrial quality control [101,102,103,104]. Loss of Irgm1 results in the accumulation of dysfunctional mitochondria, triggering the release of mitochondrial nucleic acids and the spontaneous activation of innate immune pathways. Interestingly, the downstream consequences are highly cell type specific: stromal cells lacking Irgm1 activate cGAS-STING signaling through mtDNA leakage, whereas macrophages preferentially release mitochondrial RNA and activate TLR7-dependent responses [101]. These findings illustrate how individual ISGs act in a cell-specific manner to preserve organelle homeostasis, thereby preventing inappropriate sensing of endogenous danger signals.

A similar principle applies to nuclear receptor coactivator 7 (NCOA7). Initially characterized as an antiviral restriction factor that blocks fusion between viral and endosomal membranes [105,106], NCOA7 has recently emerged as an important regulator of lysosomal homeostasis. In pulmonary arterial endothelial cells, inflammatory stimuli induce NCOA7 expression, which enhances lysosomal activity through interactions with vacuolar ATPases to promote the clearance of pathogenic oxysterols, including 25-hydroxycholesterol (25HC) [107]. The physiological importance of this pathway is highlighted by studies showing the loss of NCOA7 aggravates pulmonary arterial hypertension in animal models and by the human polymorphism rs11154337, which associates with reduced NCOA7 and increased disease severity [107].

The enzyme cholesterol-25-hydroxylase (CH25H), which synthesizes 25HC, is itself inducible by type I IFN and exerts antiviral and antibacterial cell-intrinsic immunity by altering membrane accessible cholesterol [108,109]. This type of host-directed effector function confers broad antimicrobial protection; however, it may quickly become maladaptive if persistent activation alters the functional states of target cells. Specifically, 25HC is promiscuous in its ability to bind diverse host protein targets, including SREBP2, mTORC1 and AMPKa, ADP-ribosylation factor 4 (ARF4), NLRP3, OSBP, and membrane-embedded integrins [110,111,112,113,114,115]. Allosteric regulation of each protein target is linked with a specific downstream anti-inflammatory or pro-inflammatory output, along with a multifarious assortment of inflammation-linked diseases like atherosclerosis, cancer and autoimmunity [116]. Collectively these studies posit that ISGs whose products modify cellular levels of 25HC may serve critical roles in effectuating IFN-induced changes in membrane dynamics that underlie protective or pathogenic responses depending on the tissue and disease context.

Many ISGs encode for proteins whose primary function is to curtail inflammation. Therefore, IFN signatures whose composition is selectively enriched in these negative regulators could benefit the host through their collective anti-inflammatory actions. This includes well-known signaling regulators like the suppressors of cytokine (SOCS) proteins [117], but also ISG-encoded enzymes which exert their effects by controlling levels of key cellular metabolites. One example is the IFN-inducible SAM- and HD-domain-containing protein SAMHD1, first identified as an antiviral factor that impedes infection by depleting the intracellular pool of deoxynucleoside triphosphates (dNTPs) [118,119,120]. By limiting dNTP availability, SAMHD1 also acts to restrain mitochondrial DNA synthesis and thereby opposes the activity of CMPK2 which, as discussed above, promotes the generation of dNTP precursors required for mtDNA synthesis. This balance has important physiological consequences, as in the absence of SAMHD1, uncontrolled mtDNA synthesis fuels hyperactivation of NLRP3 and downstream obesity-driven metabolic disorders [121]. Thus, while CMPK2 and SAMHD1 are both inducible by Type I IFN and are often found co-expressed within the same IFN signature, they exert opposing effects on mtDNA homeostasis and inflammasome activity, illustrating how individual ISGs can counterbalance one another to maintain immune equilibrium.

Another prominent metabolic regulator is the enzyme cis-aconitate decarboxylase (ACOD1). Encoded by immune responsive gene 1 (*IRG1*), ACOD1 is amongst the most highly upregulated proteins in IFN-γ-activated macrophages and is well known for exerting broad antibacterial and antiviral effects by converting the Krebs cycle intermediate cis-aconitate into the immunomodulatory metabolite itaconate [122,123,124,125]. However, itaconate is increasingly being recognized as a major regulator of inflammatory metabolism. Through inhibition of TET-family dioxygenases and activation of Nrf2-dependent transcriptional programs, itaconate suppresses both NF-κB and STAT1 signaling while promoting antioxidant responses [126]. These activities can protect against severe inflammatory injury and improve survival in experimental models of endotoxemia [127]. However, IRG1 also illustrates a recurring theme throughout ISG biology: protective versus pathogenic functions are often context dependent. In the lung, itaconate enhances NLRP3 inflammasome activity and exacerbates acute inflammatory injury [128]. Moreover, inhibition of succinate dehydrogenase by endogenous levels of itaconate promotes mitochondrial RNA release and subsequent activation of type I IFN pathways [129]. Beyond inflammatory disease, elevated IRG1 expression has been linked to tumor progression and resistance to immune checkpoint blockade through suppression of cytotoxic CD8^+^ T-cell responses within the tumor microenvironment [130,131]. On the other hand, IRG1/itaconate exhibits tumor suppressive effects by inhibiting the pentose phosphate pathway and endogenous itaconate is found markedly depleted in tumors compared to non-tumor tissue [132]. The reach of itaconate extends to the regulation of energy balance, where IFN-γ activated macrophages upregulate IRG1/itaconate to inhibit thermogenesis in a paracrine manner by antagonizing uptake of the pro-thermogenic metabolite succinate into brown adipose tissue [133]. Collectively, these findings illustrate how metabolic programs initiated by a single ISG can produce markedly different outcomes depending on the tissue environment and inflammatory context in which they operate.

Whereas many protective ISGs act within the cells that express them, others exert broader effects by shaping the inflammatory environment of the surrounding tissue. This includes well-known immunosuppressive cytokines like IL-10, decoy receptors like IL-18BP, and cytokine antagonists like IL1RN [134,135,136], but also IFN-inducible enzymes like the aforementioned IRG1 which suppress inflammation by regulating metabolism. Another prominent example is indoleamine 2,3-dioxygenase 1 (IDO1), a Type II IFN-inducible enzyme that catalyzes the first and rate-limiting step of tryptophan degradation. During infection, depletion of intracellular tryptophan restricts the growth of auxotrophic pathogens such as *Chlamydia*, *Toxoplasma* and *Francisella* [137]. Given that mammalian cells are also auxotrophic for this amino acid, this antimicrobial mechanism simultaneously exerts profound immunoregulatory effects in the host, first described in the landmark study by Munn et al. (1999), which established IDO1 as essential for inducing maternal T-cell tolerance [138]. By reducing tryptophan availability and generating immunosuppressive kynurenine metabolites, IDO1 limits T-cell proliferation and promotes the differentiation of regulatory T-cell populations [139,140]. Many of these effects are mediated through activation of the aryl hydrocarbon receptor pathway, which further reinforces local immune tolerance phenotypes [141,142]. Consistent with its homeostatic role, genetic variation affecting IDO1 function is associated with increased susceptibility to type II IFN-linked autoimmune disorders, including systemic sclerosis and type I diabetes [143,144,145]. These observations highlight how an ISG can simultaneously function as a potent antimicrobial effector and as a regulator of tissue tolerance and immune restraint.

Collectively, these examples demonstrate that IFN signatures contain a substantial repertoire of homeostatic effectors that preserve organelle integrity, regulate inflammatory metabolism, and limit immune-mediated tissue damage. Importantly, the same mechanisms that protect tissues in one context may become maladaptive in another, underscoring the need to view ISGs as dynamic regulators of host physiology. The balance between pathogenic and protective ISGs within an IFN signature is therefore likely to be a major determinant of tissue-specific disease trajectory. Moreover, targeting specific ISGs as opposed to upstream IFNs could be an attractive therapy for modulating the outcome of IFN signaling without compromising hots defense or triggering systemic adverse reactions as seen with many JAK/STAT inhibitors [146,147].

## 4. Mitochondria as a Signaling Hub for Protective and Pathogenic ISGs

**Figure 2:**
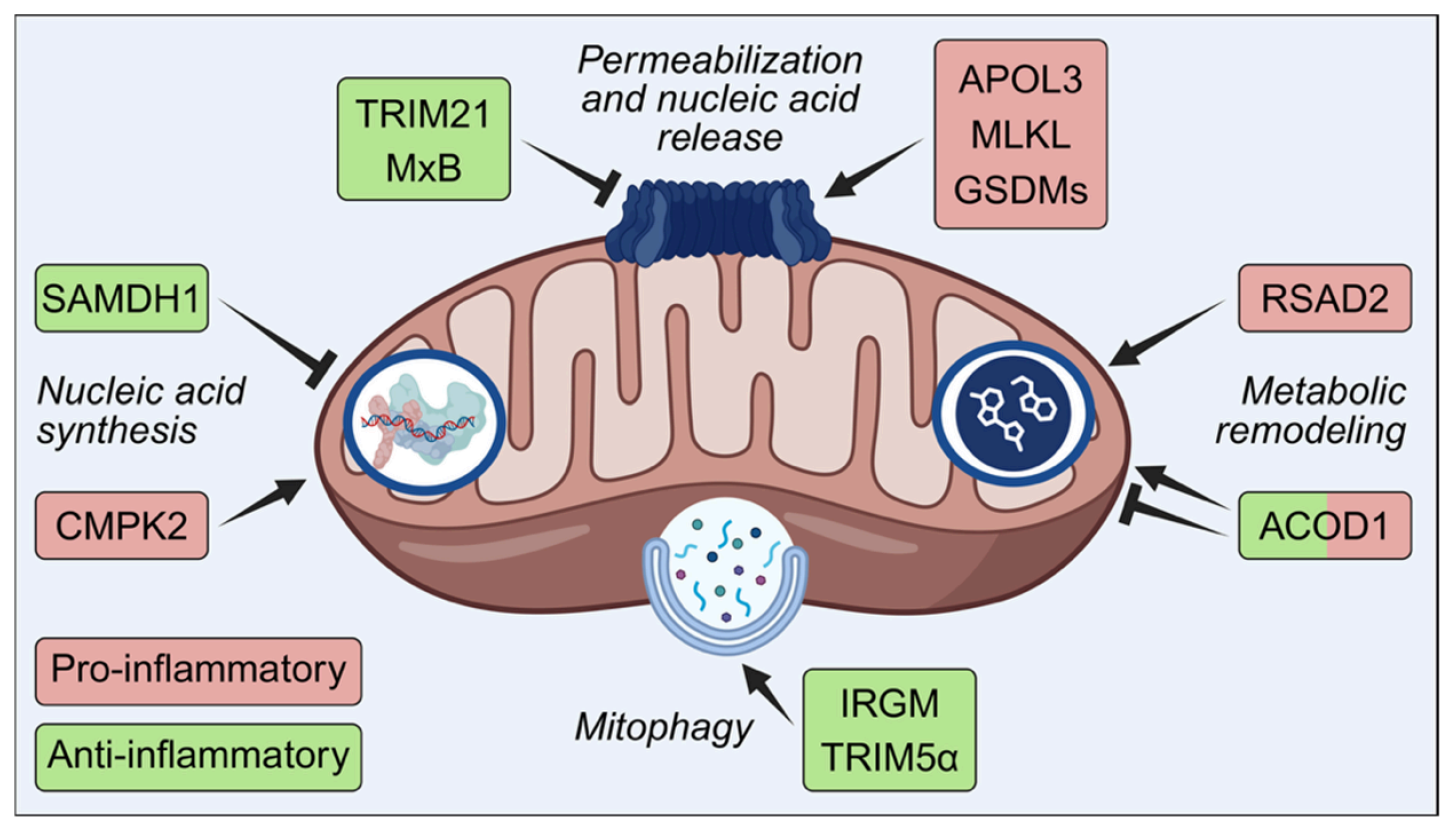
Mitochondria as signaling hub for interferon-stimulated effector functions. Summary of four major pathways through which ISG-encoded products regulate mitochondrial immunogenicity: Permeabilization to facilitate nucleic acid release; control levels of immunostimulatory mitochondrial nucleic acid synthesis; regulate mitochondrial quality control or mitophagy; and metabolic remodeling. ISGs that primarily mediate pro-inflammatory effects are indicated in red, whereas normally anti-inflammatory ISGs are depicted in green. ISGs in both red and green (e.g., ACOD1) exhibit context-dependent pro- or anti-inflammatory functions. Created in BioRender. Ritacco, D. (2026) https://BioRender.com/uydq6n5.

Numerous IFN-stimulated effectors that have been discussed herein share a common feature: despite their diverse molecular functions, they converge on the regulation of mitochondrial homeostasis. This observation suggests that mitochondria may represent a central node through which IFN-induced programs influence tissue health and disease. Traditionally recognized for their roles in energy production and cell death, mitochondria are now widely appreciated as major innate immune signaling platforms [148]. Through the regulated release of damage-associated molecular patterns (DAMPs)—which include mitochondrial DNA (mtDNA), mitochondrial RNA (mtRNA), reactive oxygen species, lipids, and cytochrome c—mitochondria serve as potent initiators of inflammatory responses. The immunological potency of these molecules likely reflects the endosymbiotic origins of mitochondria and their retention of bacterial-like features that are readily interpreted as signs of danger when cellular homeostasis is disrupted [149]. Many ISGs have therefore evolved to either promote or restrain mitochondrial immunogenicity as part of their effector mechanism (Figure 2). This framework provides a useful lens through which to interpret the seemingly disparate functions of many ISGs. Disease-linked proteins discussed previously—including IRGM, ACOD1, CMPK2, and SAMHD1—all exert major effects on mitochondrial biology despite being discovered through studies of antimicrobial immunity. Recent studies further support this idea by identifying additional ISGs whose products directly target mitochondria to either enhance or restrain immunogenicity. Although definitive evidence supporting a direct role in human disease is lacking, the ISGs discussed below illustrate how mitochondrial-directed activities may help determine whether IFN responses promote inflammation, preserve tissue homeostasis, or contribute to disease pathology.

### 4.1. ISGs That Promote Mitochondrial Immunogenicity

Several ISGs function by enhancing the ability of mitochondria to generate inflammatory signals. Though this can strengthen antimicrobial immunity, it also creates opportunities for pathological inflammation when these pathways become chronically activated. One example is the primate-specific ISG *APOL3*. Originally characterized as an antibacterial effector that selectively permeabilizes the inner membrane of cytosol-invasive Gram-negative bacteria, APOL3 can be redirected toward mitochondria under conditions of sterile lysosomal damage [150]. In this setting, APOL3 interprets the host mitochondrion as a foreign bacterium, and permeabilizes the inner mitochondrial membrane to promote mtDNA efflux and license downstream activation of the cGAS-STING pathway. During infection, this mechanism amplifies protective Type I IFN responses against viral pathogens that disrupt membrane integrity. However, lysosomal injury is not unique to infection. Environmental toxins, protein aggregates, oxidized lipids, cellular senescence, and age-associated stress all promote lysosomal leakage [151,152,153,154,155,156,157,158,159,160,161]. Consequently, APOL3 may provide a mechanistic link through which chronic tissue damage is translated into sustained IFN production. Importantly, APOL3 also illustrates the importance of maintaining segregation between distinct IFN programs. APOL3 is preferentially induced by Type II rather than by the Type I IFNs generated downstream of mtDNA sensing. This arrangement prevents the formation of a self-sustaining inflammatory circuit. Failure to maintain such compartmentalization, as may occur during aging or chronic inflammatory disease, could facilitate pathological amplification of the IFN response.

The antiviral effector viperin (*RSAD2*) provides a second example of mitochondrial remodeling in support of immunity. Although best known for generating the antiviral nucleotide analogue ddhCTP [162,163], viperin may also relocate from the endoplasmic reticulum to mitochondria where it influences both innate immune signaling and cellular metabolism. Mitochondrial viperin enhances MAVS-dependent antiviral responses while simultaneously inhibiting fatty acid β-oxidation through an interaction with the mitochondrial trifunctional protein [164,165,166]. The resulting reduction in ATP and NADPH leads to disassembly of the actin cytoskeleton and broad metabolic reprogramming characterized by AMPK activation, increased glucose uptake, enhanced lipogenesis, and lipid droplet accumulation [167]. These findings suggest that viperin acts not merely as a direct antiviral effector but as a regulator of mitochondrial metabolism that reshapes cellular physiology during the IFN response. It remains unclear what endogenous stimuli trigger the redistribution of viperin to mitochondria in the absence of infection. Answering this question will be key to understanding how the metabolic effects of viperin contribute to the pathophysiology of autoimmune diseases like Aicardi–Goutières syndrome, dermatomyositis, rheumatoid arthritis, systemic lupus erythematosus, systemic sclerosis, and Sjögren’s syndrome where viperin has been a longstanding biomarker [168,169,170,171,172,173,174,175].

Cell death effectors provide another route through which ISGs enhance mitochondrial immunogenicity. Both mixed-lineage kinase domain-like protein (MLKL) and members of the gasdermin family are induced by IFNs in a cell-specific manner and can directly target mitochondria upon their activation. While traditionally viewed as plasma membrane pore-forming proteins, both classes of effectors also generate pores within mitochondrial membranes, promoting the release of mitochondrial DAMPs that amplify inflammatory signaling [176,177]. The specific type of stressor distinguishes which effector targets mitochondria in a given cell type: MLKL is activated by necroptosis signals (TNFα + pan-caspase inhibition) or age-related replicative stress, whereas GSDMs are activated by apoptosis signals, pyroptosis activators, or neurotoxic stimuli. A second distinction lies in whether downstream inflammation occurs in a cell-autonomous or non-cell-autonomous manner. MLKL permeabilization of mitochondria encourages mtDNA release and cell-autonomous cGAS/STING activation, whereas GSDMs are primarily known for releasing a subset of extracellular DAMPs like IL-1 family cytokines and other molecules like HMGB1 that lack a secretion signal. The biological significance of mitochondrial targeting may lie in its resistance to repair. Plasma membrane damage caused by MLKL or gasdermins can be counteracted by ESCRT-dependent repair systems, whereas comparable mechanisms are absent from mitochondria [178,179]. Consequently, mitochondrial permeabilization likely generates earlier and more persistent inflammatory signals than plasma membrane damage alone. This distinction may help explain how chronic, sub-lethal activation of these pathways contribute to subtle disease phenotypes such as neurodegenerative axon loss or age-associated hematopoietic dysfunction [180,181]. A complicating factor for understanding these effectors, as is the case with many encoded by ISGs, is that a baseline of composite signals governing effector induction is incomplete, particularly given that many non-canonical signals upregulate these proteins across lymphoid and non-lymphoid tissues. For example, the *GSDM* locus is responsive to TGFβ, IRF2 activation, TNFα, and corticosteroids, each of which may co-occur with IFNs in specific inflammatory environments [182,183,184,185]. Accordingly, defining the tissue-specific activation thresholds for each effector will be essential for functionally interpreting their presence within an IFN signature.

### 4.2. ISGs That Preserve Mitochondrial Integrity

Counterbalancing these pro-inflammatory pathways is a second group of ISGs that function to maintain mitochondrial homeostasis and prevent inappropriate immune activation. As discussed above, IRGM represents one such protective factor that, through modulating mitophagy, limits the accumulation of dysfunctional mitochondria and prevents the release of immunostimulatory nucleic acid. Several classical antiviral effectors share similar functions and have recently been recognized as key regulators of mitochondrial quality control. MxB, best known for restricting HIV and herpesvirus replication [186,187], localizes to the mitochondrial matrix, where it preserves mitochondrial integrity and function [188]. Likewise, TRIM5α, originally characterized as a retroviral restriction factor [189,190], facilitates the assembly of mitophagy machinery to guard against the accumulation of mitochondrial damage [191,192]. The related protein TRIM21, an IFN-inducible cytosolic Fc receptor that controls a variety of bacterial and viral infections [193], exerts a complementary function by suppressing mtDNA release through degradation of the voltage-dependent anion channel (VDAC), thereby limiting activation of the cGAS-STING pathway [194]. The physiological significance of this activity is highlighted by observations that elevated TRIM21 expression antagonizes antitumor immunity in cancers that rely on mitochondrial DNA release to stimulate type I IFN-dependent immune responses [195]. These findings reveal an emerging theme: antiviral restriction factors often moonlight as regulators of mitochondrial quality control. In doing so, they protect tissues from excessive inflammation generated by endogenous mitochondrial danger signals.

Taken together, these observations illustrate the critical role of host mitochondria in shaping the functional output of an IFN signature. Some ISGs—including *APOL3*, *RSAD2*, *MLKL*, *GSDM*s, *IRG1*, and *CMPK2*—enhance mitochondrial immunogenicity by promoting DAMP release or facilitating downstream inflammatory signaling. Others—including *IRGM*, *SAMHD1*, *MXB, TRIM5α*, *TRIM21* and *IRG1* in certain contexts—act to preserve mitochondrial integrity and suppress inappropriate activation of innate immune pathways. The balance between these opposing activities is likely to vary substantially across tissues and disease states. Baseline cell type- and tissue-specific differences in mitochondrial abundance, metabolic activity, mtDNA content, and susceptibility to stress may further influence how individual ISGs shape cellular responses [196,197,198]. Viewed through this lens, IFN signatures are not merely collections of antimicrobial and signaling effectors but coordinated programs that determine how mitochondria respond to stress (Figure 2). The relative abundance of mitochondrial-protective and mitochondrial-disruptive ISGs may therefore represent a fundamental principle governing whether IFN signaling resolves or perpetuates inflammatory disease.

## 5. Disease-Relevant ISG Activities Revealed Through Mendelian Genetics of Type I Interferonopathies

Several of the studies discussed thus far have enlisted animal models to provide a direct link between the presence of an ISG and disease progression. However, it is important to acknowledge that limitations exist in extrapolating ISG functions from animals to humans, especially considering that ISGs are amongst the fastest evolving genes in the genome [199]. Perhaps the best evidence for ISGs exerting casual roles in disease comes from human genetics where patients with specific ISG mutations develop type I interferonopathies featuring of dysregulation and persistence of the IFN signal. These experiments of nature have revealed certain ISGs are strongly linked to the development of type I interferonopathies across a remarkably broad phenotypic spectrum [200]. Two broad lines of evidence have emerged from the genetic dissection of type I interferonopathies that emphasize the importance of individual ISGs in controlling disease trajectory. The first comes from causal loss-of-function or dominant-negative mutations in protective ISG-encoded effectors, including *PSMB8*, *ADAR1*, *ISG15*, *USP18*, *TREX1*, *SAMHD1* that lead to aberrant IFN signaling and highlight the critical regulatory roles for ISGs in curtailing excessive inflammation [201,202,203,204,205,206,207]. The other concerns gain-of-function mutations in potentially pathogenic ISGs, including *IFIH1* and *DDX58* that trigger excessive type I IFN signaling and drive pathogenicity [208,209]. Collectively, these studies provide in vivo validation that ISGs exert essential regulatory roles for balancing protective versus pathogenic inflammation in humans.

## 6. Endogenous Functions for ISGs: An Evolutionary Cost–Benefit Framework

The examples discussed throughout this perspective highlight how many ISGs exert their effects not only by directly targeting pathogens but by remodeling endogenous cellular processes. This strategy offers several important evolutionary advantages. By rewiring host physiological responses rather than targeting pathogen-derived structures, these ISGs exert broad-spectrum protection against diverse pathogens and are inherently more resistant to evolutionary escape than defenses that rely on recognition of specific microbial molecules. However, these benefits come with an important cost. Because these ISGs modify endogenous pathways that are essential for normal cellular function, their activities must be tightly regulated to prevent collateral damage. The same mechanisms that enhance host defense during infection can become harmful when inappropriately activated, persist for prolonged periods, or act in tissues particularly sensitive to their effects. IFITM3 provides a useful illustration of this trade-off. Through its ability to alter membrane properties such as lipid packing, curvature, and membrane tension, IFITM3 functions as a potent broad-spectrum antiviral factor that restricts the entry of numerous enveloped viruses. Yet, these same membrane-remodeling activities also affect host proteins. During chronic neuroinflammation, sustained IFITM3 expression enhances γ-secretase activity, promotes amyloid precursor protein processing, and accelerates amyloid plaque formation. Thus, a mechanism that likely evolved to defend against infection can become pathogenic when deployed in a different biological context [210].

The existence of such risks suggests that host organisms may have evolved safeguards to limit self-inflicted damage from ISG activity. One example is the IFN-γ-inducible kinase PIM1, which protects host membranes from the antimicrobial effector GBP1 [211]. Through phosphorylation-dependent inhibition of GBP1 GTPase activity, PIM1 prevents inappropriate targeting of self-membranes while preserving the ability to rapidly unleash GBP1-mediated defense when pathogens disrupt upstream IFN signaling. Although relatively few examples of these regulatory circuits have been described to date, they raise the intriguing possibility that ISG signatures contain built-in protective mechanisms, analogous to bacterial toxin–antitoxin systems, to balance antimicrobial efficacy against the risk of host injury.

**Figure 3:**
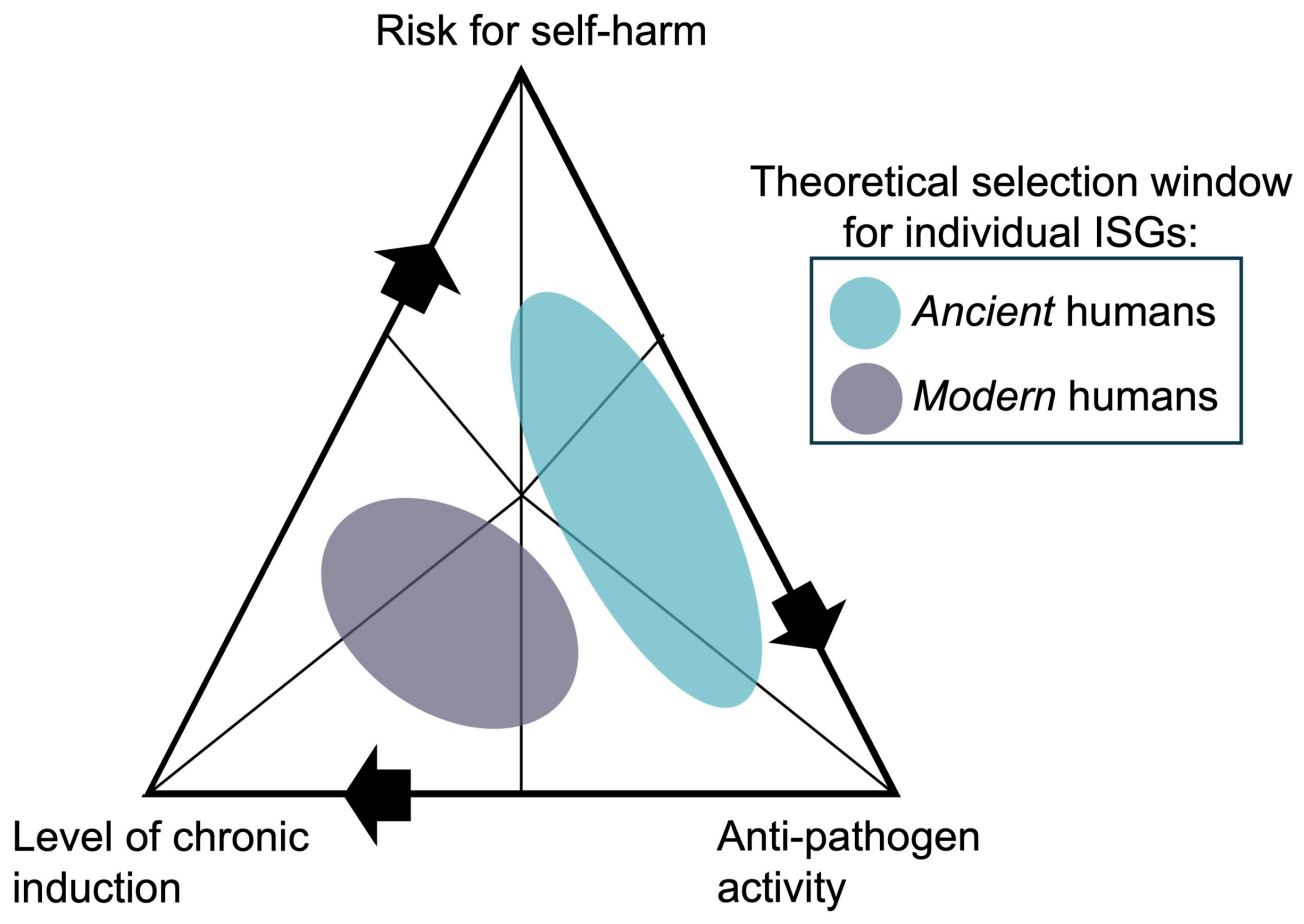
Hypothesis to explain selective pressures governing ISG evolution across human history. Ancient humans experienced strong selection for anti-infective ISGs, exerting tight regulatory control to balance the risk for self-harm. In the current environment, elevated chronic inflammation coupled with a reduced incidence of infections alters this balance by prioritizing ISGs with a lower-risk for self-harm relative to their anti-pathogen potential, or by driving the evolution of ancillary ISG functions designed to safeguard homeostasis.

Viewed through an evolutionary lens, the persistence of an ISG across time ultimately reflects a balance between the benefits it provides during infection and the costs associated with collateral tissue damage. Strong antimicrobial defenses have historically offered substantial fitness advantages in environments characterized by frequent exposure to infectious disease. In modern human populations, however, this balance may be changing. Improvements in sanitation, vaccination, antibiotics, and public health have dramatically altered infectious disease pressures. Conversely, chronic inflammation is increasingly prevalent and is now universally associated with diseases of affluence and extended lifespan like obesity, neurodegeneration and cardiovascular diseases [212]. We hypothesize that under these conditions, some ISGs may no longer operate within the environment in which they originally evolved (Figure 3).

One potential consequence of this shift is that the effectors encoded by ISGs, which individually tend to be redundant for infection resistance [213,214], may represent cases of evolutionary mismatch and be particularly susceptible to loss-of-function mutation or pseudogenization. This would manifest initially in ISGs whose primary activities carry a high risk of inflammatory self-harm. The effector APOL3 provides a potential example. APOL3 functions as an antibacterial protein but can also permeabilize host mitochondria following lysosomal damage, promoting mtDNA release and inflammatory signaling. Recent population genetic analyses identified a loss-of-function *APOL3* polymorphism undergoing positive selection in African populations [215]. Although the selective forces driving this phenomenon remain uncertain, one potential interpretation is that reduced levels of APOL3 may confer protection in environments where the inflammatory costs of mitochondrial damage outweigh the benefits of enhanced antibacterial defense. Ancestral pathogen pressures may have rendered APOL3 redundant for protection, leading to the selection for a loss-of-function allele with a lower risk for inflammatory self-harm. Consistent with this possibility, APOL3 and related family members have been implicated in a growing number of inflammatory disorders, including kidney disease, atherosclerosis, schizophrenia, and cancer [216,217,218,219,220,221,222,223,224].

The inverse scenario may also occur. As selective pressures associated with chronic inflammation increase, ISGs that preserve tissue integrity may acquire enhanced importance or evolve additional homeostatic functions. Under this model, antimicrobial activities represent only one dimension of ISG biology, whereas regulation of cellular homeostasis becomes an increasingly important determinant of fitness. A prediction arising from this framework is that loss-of-function mutations in ISGs that restrain inflammation or preserve organelle integrity should be disproportionately associated with disease susceptibility. Variants in IRGM provide a compelling example. Although IRGM contributes to antimicrobial defense, its critical role in autophagy and mitochondrial quality control appears important for maintaining intestinal homeostasis [97]. Accordingly, genetic variation affecting IRGM function is strongly associated with Crohn’s disease and other inflammatory disorders [98,99,100]. We predict that future large-scale population-based genetic studies will identify additional ISG mutations that exhibit signatures of positive selection which can be functionally tested in cellular assays for either enhanced or dampened effector functions.

Taken together, these observations support a broader view of ISG evolution in which antimicrobial activity and tissue homeostasis are not distinct functions but interconnected outcomes of the same host-defense strategies. The pathological and protective effects of ISGs described throughout this perspective may therefore reflect different manifestations of a common evolutionary principle: mechanisms that evolved to defend the host from infection are continually being repurposed, refined, and balanced against the competing need to preserve tissue function.

## 7. Conclusions

Interferons and their inducible gene signatures are amongst the most consistent molecular features associated with human disease. But despite decades of research, the specific cause-and-effect relationship remains unresolved. Do ISG signatures simply reflect tissue stress and inflammation, or do they actively shape disease progression? The examples discussed throughout this perspective support a model of mutual causation in which noxious stimuli, including infection, injury, or stress, trigger inflammation and activate ISGs, which in turn antagonize or reinforce the inflammatory response—the latter occurs at the expense of normal tissue function, whereas the former acts to restore homeostasis.

A recurring theme emerging from these studies is the central role of mitochondria as an integration point for IFN-induced responses. Many pathogenic ISGs promote the release of mitochondrial danger signals or enhance mitochondrial immunogenicity, whereas protective ISGs preserve mitochondrial integrity and limit inappropriate innate immune activation. The balance between these opposing activities may represent a fundamental determinant of how tissues respond to inflammatory stress and could help explain the remarkable tissue specificity observed across IFN-associated diseases.

Moving forward, understanding the biological significance of IFN signatures will require a shift from descriptive to mechanistic approaches. Several key challenges remain. First, we need a comprehensive understanding of which ISGs are expressed within specific cell types, tissues, and organs, and how these baseline programs differ across IFN classes. Clearly identifying whether inflammation-induced induction of specific ISGs is linked to disease in relevant tissue sites will be an important early benchmark. Second, it will be necessary to determine how disease-associated signals reshape the composition of these signatures and alter their functional output. Third, greater attention must be paid to the endogenous targets of ISGs and how their activities intersect with tissue-specific pathways that govern homeostasis. Fourth, there will be a need to incorporate proteomic approaches to address post-translational regulatory mechanisms that are also known to govern ISG functions in certain context [225]. Finally, future studies must move beyond individual effectors toward understanding how entire ISG networks function collectively, as the biological consequences of IFN signaling are likely to emerge from interactions among multiple co-expressed genes rather than any single ISG alone.

This perspective is focused primarily on how ISGs mediate the pathogenic or protective outputs of conventional IFN types (IFN-αs IFN-β and IFN-γ), given their well-established role in modulating disease outcome in mammals and the numerous lines of evidence, including many examples discussed herein, linking specific ISG-encoded effectors to these outcomes. It is also important to understand how ISGs function downstream of epithelial-restricted IFN types like IFN-λ, IFN-κ, or IFN-ε that act at barrier tissues. These IFN types are inherently less inflammatory; thus, it is tempting to speculate that they mobilize unique ISG signatures enriched in protective, or homeostatic effectors compared with more inflammatory IFNs. However, given that these IFNs are more recently discovered and exhibit more targeted and muted responses in vivo, support for individual ISGs driving their effects through endogenous processes has so-far been limited. This is an important area of future study addressed in an excellent recent review [226].

Ultimately, the IFN signature should be viewed not as a passive biomarker of inflammation, but as a dynamic regulatory program capable of reshaping tissue physiology. Defining how individual ISGs cooperate to influence homeostasis, immunity, and disease will provide a more complete understanding of IFN biology and may reveal new opportunities for therapeutic intervention across a broad spectrum of inflammatory disorders.

## Data Availability

Data generated by meta-analysis is available in Supplementary File S1. No original data were createdin this study. Data sharing is not applicable to this article.

## Supplementary Materials

The following supporting information can be downloaded at: https://www.mdpi.com/article/10.3390/biology15212018/s1, Supplementary File S1, which includes Table S1: Interferome Studies; Table S2: Interferome Genes; Table S3: Disease Studies; Table S4: Disease Effect Rank; and Table S5: Software Used.

## Abbreviations

The following abbreviations are used in this manuscript:

ISG	Interferon-stimulated Gene
IFN	Interferon
EAE	Experimental Autoimmune Encephalomyelitis
MHC	Major Histocompatibility Complex
CNS	Central Nervous System
IBD	Inflammatory Bowel Disease
SLE	Systemic Lupus Erythematosus
mtDNA	Mitochondrial DNA
IRG	Immunity-related GTPase
SOCS	Suppressors of Cytokine Signaling
dNTP	Deoxynucleoside Triphosphates
DAMP	Damage-associated Molecular Patterns
AGS	Aicardi–Goutières Syndrome
HIV	Human Immunodeficiency Virus
VDAC	Voltage-dependent Anion Channel
